# Screening of male breast cancer and of breast-ovarian cancer families for *BRCA2* mutations using large bifluorescent amplicons

**DOI:** 10.1054/bjoc.2000.1627

**Published:** 2001-02

**Authors:** S Pages, V Caux, D Stoppa-Lyonnet, M Tosi

**Affiliations:** Service de Génétique Oncologique, Institut Curie, 26 rue d'Ulm 75248, Paris Cedex 5; Unité d'Immunogénétique et INSERM U 276, Institut Pasteur, 25 rue du Docteur Roux, Paris, 75724, Cedex 15; INSERM EMI 9906, Faculté de Médecine et Pharmacie, 76183 Rouen, France

**Keywords:** male breast cancer, breast-ovarian cancer, FAMA, chemical cleavage of mismatch, chimeric PCR primers

## Abstract

41 breast cancer or breast-ovarian cancer families, including 12 families with at least one affected first-degree male relative, were screened for mutations in the *BRCA2* gene. Mutations had not been found in the *BRCA1* gene of these families. Chemical cleavage of Mismatch was used to identify nucleotide changes within large PCR products (average size 1.2 kb) that carried strand-specific fluorescent end-labels. 15 amplicons were sufficient to scan 18 exons, including the large exon 11. The remaining 9 small exons were examined by Denaturing Gradient Gel Electrophoresis. The high sensitivity of this approach was documented by the detection, in these 41 patients, of all 9 exonic single nucleotide polymorphisms reported with heterozygosity >0.1. Truncating *BRCA2* mutations were found in 7 of the 41 families. 3 of them were in the group of 12 families comprising cases of male breast cancer. Since the methods used here have no bias for particular types of mutations, these data confirm the high proportion of frameshifts among mutations in *BRCA2*. However, relevant single nucleotide substitutions were also found: one resulting in a stop codon and another one, present in a male patient, was the previously reported change Asp2723His, that affects a highly conserved region of the BRCA2 protein. This study indicates a *BRCA2* contribution of 10% (95% CI 2.5–17.5) to our original cohort of 59 breast-ovarian cancer families, whereas the contribution of *BRCA1* had been estimated at 46% (95% CI 33–59). © 2001 Cancer Research Campaign http://www.bjcancer.com


*British Journal of Cancer* (2001) **84**(4), 482–488
© 2001 Cancer Research Campaign
doi: 10.1054/ bjoc.2000.1627, available online at http://www.idealibrary.com on IDEAL®

http://www.bjcancer.com

### Screening of male breast cancer and of breast-ovarian cancer families for *BRCA2* mutations using large bifluorescent amplicons

**S Pages[1], V Caux[1], D Stoppa-Lyonnet[1] and M Tosi[2,3]**

[1]Service de Génétique Oncologique, Institut Curie, 26 rue d'Ulm 75248 Paris Cedex 5; [2]Unité d'Immunogénétique et INSERM U 276, Institut Pasteur, 25 rue du Docteur Roux, 75724 Paris, Cedex 15; [3]INSERM EMI 9906, Faculté de Médecine et Pharmacie, 76183 Rouen, France

**Summary** 41 breast cancer or breast-ovarian cancer families, including 12 families with at least one affected first-degree male relative, were screened for mutations in the *BRCA2* gene. Mutations had not been found in the *BRCA1* gene of these families. Chemical cleavage of Mismatch was used to identify nucleotide changes within large PCR products (average size 1.2 kb) that carried strand-specific fluorescent end-labels. 15 amplicons were sufficient to scan 18 exons, including the large exon 11. The remaining 9 small exons were examined by Denaturing Gradient Gel Electrophoresis. The high sensitivity of this approach was documented by the detection, in these 41 patients, of all 9 exonic single nucleotide polymorphisms reported with heterozygosity >0.1. Truncating *BRCA2* mutations were found in 7 of the 41 families. 3 of them were in the group of 12 families comprising cases of male breast cancer. Since the methods used here have no bias for particular types of mutations, these data confirm the high proportion of frameshifts among mutations in *BRCA2*. However, relevant single nucleotide substitutions were also found: one resulting in a stop codon and another one, present in a male patient, was the previously reported change Asp2723His, that affects a highly conserved region of the BRCA2 protein. This study indicates a *BRCA2* contribution of 10% (95% CI 2.5–17.5) to our original cohort of 59 breast-ovarian cancer families, whereas the contribution of *BRCA1* had been estimated at 46% (95% CI 33–59). © 2001 Cancer Research Campaign http://www.bjcancer.com

**Keywords**: male breast cancer; breast-ovarian cancer; FAMA; chemical cleavage of mismatch; chimeric PCR primers

The relative contribution of the two major breast cancer predisposing genes, *BRCA1* and *BRCA2*, differs in breast cancer-only families and in breast-ovarian cancer families (reviewed in Rahman and Stratton, 1998). While *BRCA1* defects are found in a majority of high-risk breast-ovarian cancer families, defects in the second breast cancer predisposition gene, *BRCA2* (Wooster et al, 1995) are involved in a large fraction of high-risk families with at least one case of male breast cancer (Ford et al, 1998). In July 2000, the Breast cancer information core (BIC) reports close to 900 *BRCA2* sequence variants, of which about half are truncating mutations or splicing defects that are likely to determine RNA instability or protein truncation. These changes are distributed over the entire coding sequence (10254 nt) and exon–intron boundaries.

The aim of this study was threefold: (1) to adapt to *BRCA2* a protocol that allows sensitive and unbiased detection of all types of mutations using a small number of large amplicons; (2) to describe the contribution of *BRCA2* mutations in French male breast cancer families; (3) to estimate the contribution of *BRCA2* mutations in breast-ovarian cancer families.

Fluorescence-Assisted Mismatch Analysis (FAMA) (Verpy et al, 1994, 1996) was chosen as the most appropriate method for mutation detection. It relies on detection of mismatches in heteroduplex DNA which carries strand-specific fluorophores and allows sensitive detection and precise positioning of mutations within large amplicons (average size 1.2 kb). Therefore the cost and time involved in sequencing and in the analysis of sequence data are strongly reduced. The structure of *BRCA2* (Wooster et al, 1995; Tavtigian et al, 1996) lends itself to scanning with large amplicons, because of the large size of several exons and also because several clusters of exons can be amplified within large PCR products, when relatively small introns are present.

*Received 24 July 2000*
*Revised 18 October 2000*
*Accepted 8 November 2000*

*Correspondence to:* M Tosi, INSERM EH19906, Faculté de Médecine et Pharmacie, 76183 Roven, France

### PATIENTS AND METHODS

### Patients

The *BRCA2* gene was screened in two groups of patients. Group 1 consisted of 12 families comprising at least two cases of breast cancer and at least one affected first-degree male relative. Of these 12 families, 3 also presented one or more ovarian cancer cases. The mutational status was known in one of the latter families (IC20, Tavtigian, 1996). Group 2 consisted of 29 breast ovarian cancer families. The inclusion criteria were: at least one case of ovarian cancer in a women with a first-degree relative affected by breast or ovarian cancer.

All cases reported here were from a consecutive series of patients ascertained in our genetic counseling sessions at the Institute Curie, Paris between 1994 and 1997. All 41 index cases had been found negative for point mutations or micro-deletions/insertions upon examination of the *BRCA1* gene by scanning all exons and exon–intron boundaries using denaturing gradient gel electrophoresis (DGGE; Stoppa-Lyonnet et al, 1997; and our unreported data). The probability of being a mutation carrier at a general autosomal dominant breast cancer susceptibility locus was calculated taking into account all the individual and familial information and using the parameters of the genetic model of the Breast Cancer Linkage Consortium study (Easton et al, 1993).

### Mutation scanning strategy

The FAMA scanning primers (Table 1 and Figure 1) were selected according to the following criteria: 1. amplicons should be in the 1.0 to 1.4 kb range, with overlaps of at least 150 bp, if necessary; 2. the 3′ end of primers should correspond to a rare sequence. To this end, the octamer frequency disparity criterium was used (Griffais et al, 1991) with the software PC-rare, available from http://bioinformatics.weizmann.ac.il/pub/software/; 3. chimaeric primers were designed by extending the 5′ end of gene-specific sequences with universal rare 16-mers that differ for the forward and reverse primers (Table 1). This protocol allows economical strand-specific end-labelling by using, in a second PCR, a single universal set of fluorescent primers. Primers used for the 9 small exons scanned by DGGE are listed in Table 2.

### Polymerase chain reactions for fluorescent chemical cleavage of mismatch

250 ng of genomic DNA isolated from peripheral white blood cells was used for 25 cycles of PCR amplification in a 20 μl reaction with 6 pmoles of chimeric primers. The latter were designed by extending the gene-specific sequence with ‘universal’ 16-mers, added to their 5′ end and differing for the forward and the reverse primers (see Table 1). PCR conditions were: denaturation at 94˚C for 5 min followed by 25 cycles at 94˚C for 30 sec, X˚C for 45 sec, 72˚C for 2 min (where X is the annealing temperature given in Table 1) and final extension at 72˚C for 5 min. Amplitaq (Perkin Elmer) was used (0.5 units in 25 μl) with standard PCR buffer (1.5 mM $MgCl_2$, 250 uM dNTPs). The fluorescent template was generated by reamplifying, for 25 cycles, 1 μl of the first amplification reaction using universal fluorescent primers end-labelled with 6-FAM & HEX (Perkin-Elmer/ABD), respectively. Heteroduplexes were formed directly after reamplification, by adding to the PCR program a denaturation step at 98˚C for 10 min and a renaturation step at 60˚C for 40 min. Correct amplification was tested on 0.8% agarose gels. Bichrome PCR fragments were ethanol precipitated in a dry ice/ethanol bath, upon addition of 60 ug of glycogen carrier (Boehringer, Mannheim) and resuspended in 18 μl of 10 mM Tris, pH 8.5. 6 μl of DNA was treated at 37˚C for 30 min with 20 μl of 7 M hydroxylamine hydrochloride and another aliquot of 6 μl was incubated for 15 min at 15˚C in 0.4% osmium tetroxide/2% pyridine/5 mM Hepes, pH 8.0/0.5 mM $Na_2$EDTA in a total volume of 25 μl. Aliquots of 7 M hydroxylamine hydrochloride (Merck) solution, titrated to pH 6.0 by addition of diethylamine (Fluka), were stored at –80˚C. Osmium tetroxide (Aldrich, 4% (wt/vol) in water) was diluted in distilled water to give a 1% stock solution, aliquoted and stored at –80˚C. Mixes were prepared on ice for the osmium tetroxyde reaction and at room temperature for the hydroxylamine reaction. Modification reactions were terminated by transferring the samples to ice and by adding 200 μl of 0.3 M sodium acetate/0.1 mM $Na_2$EDTA, pH 5.2 and the nucleic acids were ethanol precipitated twice. Pellets were resuspended in 50 μl of 1 M piperidine (Aldrich) and incubated at 90˚C for 20 min. 5 μg of yeast tRNA and 50 μl of 0.6 M sodium acetate (pH 6.0) were added, and the nucleic acids were ethanol precipitated and dried. Pellets were resuspended in 8 μl of a 5/1 mixture of 100% formamide and 25 mM $Na_2$EDTA. 4 μg of each sample, mixed with 0.5 μl of fluorescent-labelled size standard (GS2500PROX; Applied Biosystems) were electrophoresed in a 4.25% acrylamide gel in a PE Applied Biosystems 377 DNA sequencer. Data were analysed using the Genescan™ software.

### Denaturing gradient gel electrophoresis (DGGE)

50 ng of genomic DNA isolated from peripheral white blood cells was used for 35 cycles of PCR amplification in a 10 μl reaction with 3 pmoles of oligonucleotide primers (Table 2). PCR conditions were: denaturation at 94˚C for 5 min followed by 35 cycles at 94˚C for 30 sec, Y˚C for 30 sec, 72˚C for 30 sec (where Y is the annealing temperature given in Table 2) and final extension at 72˚C for 5 min. To the 5′ end of one of the primers, a GC-rich sequence was added as a GC-clamp. Heteroduplexes were formed directly after the amplification, by denaturation at 98˚C for 10 min and renaturation in a 98˚C to 25˚C gradient during 40 min. 10 μl of bromophenol blue/xylene cyanol were added to the PCR products, and 20 μl were loaded on a DGGE gel. The electrophoresis conditions (for gradient and run time see Table 2) were determined by use of the Meltmap and SQHTX programs.

### DNA sequencing

Amplicons revealing the presence of sequence variants were sequenced using the Big Dye Terminator kit (PE Applied Biosystems) either entirely (for changes detected by DGGE) or around the region where the sequence change had been detected and positioned by FAMA.

### RESULTS

### Mutation detection strategy

Taking advantage of the large scanning window of the FAMA method, we have designed primers allowing amplification and fluorescent labelling of segments larger than 1 kb (Figure 1 and Table 1). FAMA exploits end-labelling of large amplicons with strand-specific fluorescent chromophores to determine, upon chemical cleavage of mismatch, not only the presence of unpaired or mispaired bases in heteroduplex DNA, but also their precise location (Verpy et al, 1994, 1996). As shown in Figure 1, exon 11 was divided into 5 amplicons with overlaps of 175 to 299 bp. Exons 10, 14 and 27 were encompassed each by a large amplicon. One should note that primers are positioned in introns at an average distance of 150 bp from the exon boundaries, because the initial stretch of about 80 bp of the fluorescent cleavage profile cannot be interpreted with confidence due to background in the lower portion of the gel. Most other exons were grouped within large amplicons. Therefore, introns 1, 5, 6, 17, 19, 22 and 23 were scanned entirely, as shown schematically in Figure 1. 9 small exons flanked by large intervening sequences, depicted in red in Figure 1, were modelled for DGGE (Table 2), because of the proven sensitivity of this method for target DNA in the range of 400 bp. Exon 15 was analysed by FAMA, in spite of its small size

*© 2001 Cancer Research Campaign* 

**Table 1** Primers used for FAMA

| Amplicon | Forward primer[a] | Reverse primer[b] | Annealing (°C) | Size (bp)[c] |
|---|---|---|---|---|
| EX 1–2 | 5′ ATTCGGTCAGAACTGACGGTTG 3′ | 5′GTGGTTAACCTGCAAACGATGAT 3′ | 56 | 1395 |
| EX 3 | 5′ GTTACACCTTTCTATAGATTCGCAA 3′ | 5′ CATCGTCTCCATTTTTCGAGTG 3′ | 64 | 617 |
| EX 5–6–7 | 5′ GAGTTTAAAATACACGGTTTCC 3′ | 5′ CTACGTTAATCACATCGTACTA 3′ | 56 | 1027 |
| EX 10 | 5′ AGAAGGGGTGACTGACCGAAA 3′ | 5′ GAAAAAAACACAGAAGGAATCGTCA 3′ | 66 | 1391 |
| EX 11.11 | 5′ GCCTCCCAAAAGTGCTGAGATTA 3′ | 5′ GAAGCTGTTCTGAAGCTACCTCC 3′ | 64 | 1297 |
| EX 11.12 | 5′ GGTTTATGTTCTTGCAGAGGAG 3′ | 5′ GCAAGTCCGTTTCATCTTTATGA 3′ | 64 | 1265 |
| EX 11.20 | 5′ GAAATGACTACTGGCACTTTTGTTG 3′ | 5′ GTATTTATTCTTTCTGGTTGACCATC 3′ | 62 | 1262 |
| EX 11.31 | 5′ GAGACTGTGGTGCCACCTAAG 3′ | 5′ GGAAAAGACTTGCTTGGTACTATC 3′ | 62 | 1251 |
| EX 11.32 | 5′ GTCTGGATTGGAGAAAGTTTCTA 3′ | 5′ GTATATCAAACCATACTCCCCCA 3′ | 64 | 1222 |
| EX 14 | 5′ TATGTGTTATGTGAGGTAGATTG 3′ | 5′ AACATTAGAATAATTTAAACCTAATC 3′ | 62 | 834 |
| EX 15 | 5′ GTCTTGAACTCCCGACCTCA 3′ | 5′ GCAGGCTAATTAGAAAATATGATG 3′ | 62 | 509 |
| EX 17–18 | 5′ CAAAATGCTGGGAGTATAGGC 3′ | 5′ GAAATTGAGCATCCTTAGTAAGC 3′ | 58 | 1383 |
| EX 19–20 | 5′ TTACTTATTTACTGTCTTACTAATC 3′ | 5′ CAAAAAAGAATACCCTAGATACTAA 3′ | 62 | 1037 |
| EX 22–23–24 | 5′ GAATATTATGTGAGAAACTGATTAC 3′ | 5′ TTACTTTCAGATCACTAGTTAGC 3′ | 62 | 1142 |
| EX 27 | 5′ TGAACTGAAATCACCTAACCTATTA 3′ | 5′ TGAAGCAAAAGTATACCAATACGG 3′ | 62 | 990 |

[a]Universal extension: 5′TAGTCGACGACCGTTA3′. Fluorescent primer: 5′-[BFAM]ggTAGTCGACGACCGTTA-3′. [b]Universal extension: 5′TCGGATAGCTAGTCGT3′. Fluorescent primer: 5′-(HEX)ggTCGGATAGCTAGTCGT-3′. [c]Including extensions.

**Table 2** Primers and DGGE conditions

| Amplicon | Forward primer | Reverse primer | Annealing (°C) | Size (bp) | Gradient (%) | Migration (h) |
|---|---|---|---|---|---|---|
| EX 4 | 5′ F(GC) CATTCTCATTCCCAGTATAGAGG 3′ | 5′ AGATCTTCTACCAGGCTCTTAGC 3′ | 58 | 340 | 20–70 | 4 |
| EX 8 | 5′ F(GC) GATTGACCTTTCTAATTACTATAC 3′ | 5′ AAATAATTTAACAAGGCATTCC 3′ | 50 | 344 | 20–70 | 2 |
| EX 9 | 5′ F(GC) GGACCTAGGTTGATTGCAGATAAC 3′ | 5′ CGGTAAACTGAGATCACGGGTGACA 3′ | 65 | 390 | 20–70 | 2 |
| EX 12 | 5′ F(GC) GGTCTATAGACTTTTGAGAA 3′ | 5′ GTCAGAATATTATATACCCATACC 3′ | 50 | 293 | 10–60 | 2 |
| EX 13 | 5′ CAGTAACATGGATATTCTCTTAG 3′ | 5′ R(GC) AGTGTCATTATTTTTAGAAATGTTC 3′ | 58 | 274 | 10–60 | 2 |
| EX 16 | 5′ F(GC) GTGTGATACATGTTTACTTTAAATTG 3′ | 5′ GTTCGAGAGACAGTTAAGAGAAG 3′ | 62 | 404 | 20–70 | 2 |
| EX 21 | 5′ GGGTGTTTTATGCTTGGTTCTTT 3′ | 5′ R(GC) ATGGCCAGAGAGTTAAAACAGC 3′ | 62 | 334 | 20–70 | 3 |
| | | | | | 30–80 | 8 |
| EX 25 | 5′ F(GC) CTTGCATCTTAAAATTCATCTAAC 3′ | 5′ GATACTGGACTGTCAAAATAG 3′ | 52 | 434 | 20–70 | 5 |
| EX 26 | 5′ F(GC) GGTCCCAAACTTTTCATTTCTGC 3′ | 5′ GTATACAACAGAATATACGATGG 3′ | 58 | 381 | 10–60 | 7 |
| | F(GC): CCCCGCCCGGCCCGCCCCGCCCCCC GCCCCCCCTCCCGGCCCGCCCCCC TGGCGCCCCGC. | R(GC): CCCCACGCCACCCGACGCCCCAGCCC GACCCCCCCGCGCCCGGCGCCCCCGC. | | | | |

(exon size: 165 bp; FAMA amplicon 509 bp), because it was not straightforward to find suitable DGGE conditions. Among the main characteristics of the FAMA method, one should note the ability to detect multiple changes within large amplicons, thanks to the limited and controlled nature of the chemical modification reactions that always result in partial cleavage. Figure 2 shows for example the profiles obtained for polymorphic sites in the 5′ end of the gene (Panel A) and for one of the truncating mutations found in this study (Panel B). The previously reported polymorphic transition 203 G→A in exon 2 precedes the translation initiation site. Panel B of Figure 2 illustrates the use of another large amplicon that encompasses exon 17, intron 17 and exon 18 and shows a single nucleotide deletion found in exon 18. The variant alleles found in this study are summarized in Table 3. The G203A variant in exon 2 is apparently in linkage disequilibrium with a previously unreported polymorphic site in intron 1 (+ 421 G→T), since this association has been observed in 13 individuals. Two additional previously undescribed polymorphic sites, apparently also in linkage disequilibrium, were found in intron 1 at positions + 164 and + 222 (Table 3). Among the coding alleles listed in Table 3, the exon 11 amino acid substitution Asn1147Ser deserves particular attention. While a contribution to cancer predisposition cannot be excluded formally, we consider this change as probably non-pathogenic because it was found in the index case of family 153, shown below, who has a truncating mutation.

### Male breast cancer families

12 apparently BRCA1-negative families with at least one case of male breast cancer were studied. As shown in Table 4, 3 truncating mutations were found (families 32, 20 and 153). In addition the missense change Asp2723His was found in family 196. In this case the *BRCA2* status of the son of the index case, who died from breast cancer, could not be determined because histology slides were refractory to PCR amplification.

Based on our finding of 3 truncating mutations and one potentially predisposing missense change, the contribution of *BRCA2* mutations to male breast cancer in this particular group of families appears to be in the 25–35% range. 3 of these 12 male breast cancer families also had one or more cases of ovarian cancer and two of the truncating *BRCA2* mutations were found in this subset (Table 4).

### Breast-ovarian cancer families

In addition, 29 breast/ovarian cancer families were studied (Table 4). In these families, 4 truncating *BRCA2* mutations were found: 3

 © 2001 Cancer Research Campaign

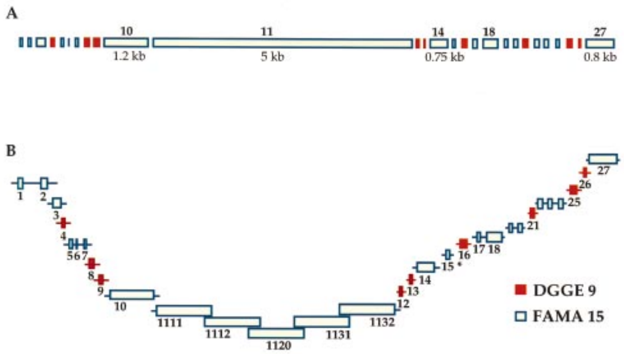

**Figure 1** Design of amplicons. (**A**) Schematic representation of the 27 exons of *BRCA2* (on scale). (**B**) Amplicons and extent of intron sequences scanned (amplicon sizes are listed in Tables 1 and 2). Regions scanned by FAMA and by DGGE are shown in green and red, respectively. The distance of the FAMA fluorophore from exon boundaries was in each case at least 150 bp. Within exon 11, amplicons 1111, 1112, 1120, 1131 and 1132 overlapped by 198, 175, 299, and 229 bp (from left to right)

in exon 11 (families 946, 1063 and 1332) and one in exon 23 (family 1006). Thus, a total of 6 truncating mutations were found in 32 breast-ovarian cancer families, including the two mutations found in families with male breast cancer cases. 3 of these 6 mutations were within the region of exon 11 defined as OCCR (ovarian cancer cluster region) by Gaither et al (1997).

As the calculated probabilities of being a mutation carrier varied considerably (Table 4), we examined the distribution of the mutations found according to the level of predisposition probability. Note that the calculation of predisposition probabilities (see the Patients and Methods section) takes into account the whole family history, including number of cases, pedigree distribution, age of disease onset and current age of unaffected relatives. Only 2 of the 8 truncating mutations were found in patients with probabilities lower than 95% (one in a male breast cancer patient and one in the breast-ovarian cancer group). Conversely, 5 of the 16 apparently *BRCA1*-negative index cases with predisposition probabilities of 95% or more had truncating mutations in *BRCA2*.

### DISCUSSION

The mutation scanning strategy described here for *BRCA2* (Figure 1) covers all exons and large segments of the intron sequences and is based on the use of a small number of amplicons (i.e. 15 large amplicons scanned with FAMA and 9 small amplicons scanned with DGGE, see Figure 1). This protocol does not require expensive reagents nor specialized equipment in addition to a DNA sequencer. Moreover, FAMA reduces substantially the number of sequencing reactions, thanks to the precise information about the position of the nucleotide change. All mutations and sequence variants reported in this study (Tables 3 and 4) were found in the regions scanned by FAMA. This result was not surprising, because FAMA covered more than 80% of the coding sequences and two-thirds of the splice site junctions, corresponding to a total of 15 kb of the *BRCA2* sequence, while only 3 kb were scanned by DGGE.

A global assessment of the sensitivity of this approach with regard to point mutations is provided by our ability to detect a large number of single nucleotide polymorphisms, as shown in Table 3. In fact, all 9 exonic single nucleotide polymorphisms that have been reported by Wagner et al (1999) with global heterozygosity of at least 0.1 were detected in this relatively small sample of 41 unrelated patients (Table 3). 3 frequent intronic polymorphic changes described in that study (introns 11, 14 and 20) have not been found in this work because our primer design in these regions was not aimed at ensuring detection of sequence changes at distances larger than 50 bp from the exon boundaries (see Figure 1). Conversely, as shown in Table 3, we found unreported polymorphic sites in intron 1 (positions 189 + 421, 189 + 222 and 189 + 164) in exon 11 (positions 4266 and 3668) in intron 22 (position 9181 + 135) and in exon 27 (positions 10589 and 10591).

As the combination of methods used here affords high sensitivity of detection, not only for frameshifts but also for single

**Table 3** Observed variants in *BRCA2*

| Exon/intron | Nucleotide | Codon | Nucleotide change | Amino acid change | Observed heterozygotes (%) | | BIC report |
|---|---|---|---|---|---|---|---|
| Intron 1 | IVS + 421 | – | G>T | non-coding | 13/82 (16) | 13 (32) | No |
| Intron 1 | IVS1 + 222 | – | C>T | non-coding | 12/82 (15) | 12 (29) | No |
| Intron 1 | IVS1 + 164 | – | A>G | non-coding | 12/82 (15) | 12 (29) | No |
| Exon 2 | 203 | – | G>A | non-coding | 13/82 (16) | 13 (32) | Yes |
| Exon 10[a] | 1093 | 289 | A>C | Asn>His | 1/82 (1) | 1 (2) | Yes |
| Exon 10 | 1342 | 372 | A>C | His>Asn | 15/82 (18) | 15 (36) | Yes |
| Exon 10 | 1593 | 455 | A>G | Ser (Silent) | 1/82 (1) | 1 (2) | Yes |
| Exon 11 | 2457 | 743 | T>C | His (Silent) | 2/82 (2) | 2 (4) | Yes |
| Exon 11[a] | 3199 | 991 | A>G | Asn>Asp | 2/82 (2) | 2 (4) | Yes |
| Exon 11 | 3624 | 1132 | A>G | Lys (Silent) | 2/82 (2) | 2 (4) | Yes |
| Exon 11[b] | 3668 | 1147 | A>G | Asn>Ser | 1/82 (1) | 1 (2) | No |
| Exon 11 | 4035 | 1269 | T>C | Val (Silent) | 6/82 (7) | 6 (14) | Yes |
| Exon 11 | 4266 | 1346 | T>C | Thr (Silent) | 1/82 (1) | 1 (2) | No |
| Exon 11 | 5972 | 1915 | C>T | Thr>Met | 3/82 (4) | 3 (7) | Yes |
| Exon 14 | 7470 | 2414 | A>G | Ser (Silent) | 1/82 (1) | 1 (2) | Yes |
| Intron 22 | IVS22 + 135 | – | C>G | non-coding | 1/82 (1) | 1 (2) | No |
| Intron 26 | IVS26 – 20 | – | C>T | non-coding | 1/82 (1) | 1 (2) | No |
| Exon 27 | 10589 | – | A>C | non-coding | 4/82 (5) | 4 (10) | No |
| Exon 27 | 10591 | – | A>C | non-coding | 5/82 (6) | 5 (12) | No |

[a]In one patient (F196) Asn289His (exon 10) and Asn991Asp (exon 11) were found together with Asp2723His (cf Table 4). [b]Asn1147Ser was found in the index case of family 153, who has a truncating mutation (cf Table 4).

© 2001 Cancer Research Campaign

**Table 4** BRCA2 mutation screening in 41 families (including 12 with male breast cancers)

| | Familial cancers[a] | | | | Index case | | BRCA2 mutations | | |
|---|---|---|---|---|---|---|---|---|---|
| | BCs | | | | | | | Nucleotide | Aminoacid |
| Family[b,d] | M | F | OC | B/OC | Sex | PP (%)[c] | Exon | change | change |
| 32 | 1 | 1 | – | | M | 43 | 10 | 1529del4 | ter460 |
| 154 | 1 | 1 | – | | M | 65 | | | |
| 196 | 2 | – | – | | M | 93 | 18 | G8395C | Asp2723His |
| 344 | 1 | 3 | – | | M | 54 | | | |
| 392 | 1 | 3 | – | | F | 93 | | | |
| 415 | 1 | 2 | – | | F | 89 | | | |
| 423 | 1 | 2 | – | | M | 87 | | | |
| 469 | 1 | 2 | – | | F | 93 | | | |
| 738 | 1 | 1 | – | | M | 74 | | | |
| 20 | 2 | 6 | 2 | | M | 98 | 18 | 8525delC | ter2776 |
| 153 | 1 | 10 | 1 | | F | 96 | 18 | 8222delA | ter2676 |
| 503 | 1 | 5 | 1 | | M | 96 | | | |
| 16 | – | 3 | 1 | | F | 97 | | | |
| 51 | – | 2 | – | 1 | F | 90 | | | |
| 301 | – | 1 | 2 | | F | 85 | | | |
| 557 | – | – | 1 | 1 | F | 90 | | | |
| 659 | – | 5 | 1 | | F | 95 | | | |
| 707 | – | 4 | 1 | | F | 98 | | | |
| 827 | – | 1 | 2 | | F | 96 | | | |
| 842 | – | 2 | 2 | | F | 96 | | | |
| 887 | – | 3 | 1 | | F | 90 | | | |
| 901 | – | 3 | – | 1 | F | 80 | | | |
| 902 | – | 2 | 1 | | F | 97 | | | |
| 946 | – | 3 | 1 | | F | 93 | 11 | 3970del4 | ter1258 |
| 964 | – | 1 | 1 | | F | 33 | | | |
| 1006 | – | 2 | 1 | | F | 97 | 23 | 9254del5 | ter3016 |
| 1063 | – | 5 | 1 | | F | 96 | 11 | C5873A | ser1882ter |
| 1079 | – | 3 | 1 | | F | 76 | | | |
| 1083 | – | 3 | 1 | | F | 85 | | | |
| 1084 | – | 2 | 1 | | F | 93 | | | |
| 1092 | – | 3 | 1 | | F | 92 | | | |
| 1109 | – | 2 | 3 | | F | 96 | | | |
| 1139 | – | 1 | 1 | | F | 49 | | | |
| 1158 | – | 2 | 1 | | F | 96 | | | |
| 1165 | – | 1 | 2 | | F | 98 | | | |
| 1182 | – | 3 | 1 | | F | 96 | | | |
| 1208 | – | 1 | 1 | | F | 10 | | | |
| 1244 | – | 2 | 1 | | F | 67 | | | |
| 1245 | – | 3 | 1 | | F | 85 | | | |
| 1332 | – | 6 | 1 | | F | 95 | 11 | 5579delA | ter1785 |
| 1389 | – | 4 | 4 | | F | 77[e] | | | |

[a]Familial cancers: BC, breast cancer; M, male; F, female; OC, ovarian cancer; B/OC, breast/ovarian cancer. [b]The first twelve families have at least one case of male breast cancer. [c]PP, predisposition probability of the index case. [d]Underlined numbers denote families in which the index case had a predisposition probability of at least 95%. [e]Low predisposition probability because the 4 ovarian cancer cases belong to 3 different parental lineages.

nucleotide substitutions, these data strengthen the notion that truncating mutations, mainly due to frameshifts, represent the largest fraction of *BRCA2* mutations. However we found a large number of sequence variants resulting from point mutations and a potentially pathogenic missense change (Asp2723His in exon 18 of family 196; Table 4). This missense change has been reported 7 times in the BIC database as an uncharacterized variant, but has not been found in normal controls nor in a recent study of individuals from world-wide populations (Wagner, 1999). In this study it was found in the index case of a family with 2 male breast cancer cases, but other family members were not available for further studies. This amino acid substitution differs from the 5 ones, listed as coding variants in Table 3, that have been found either in normal individuals (Wagner et al, 1999) or in a patient carrying the truncating mutation 8222delA (Asn1147Ser found in family 153; see Table 4). As shown in Figure 3, Asp2723 is a conserved amino acid and the amino acid sequence encoded by exon 18 (residues 2659–2778) is also highly conserved. Moreover, the fragment of BRCA2 between amino acids 2472 and 2957 has 77% identity between mouse and man (compared with 59% overall) and is the site of an interaction with the product of the DSS1 gene (Marston et al, 1999). This region is distinct from the regions of interaction of BRCA2 with RAD51 as well as from the position of the nuclear localization signal (Welcsh et al, 2000). Based on these observations, the amino acid substitution Asp2723His might contribute to cancer predisposition and its effect on functional interactions of the BRCA2 protein should be investigated. It is of interest to note that this change was found in the index case of

*© 2001 Cancer Research Campaign*

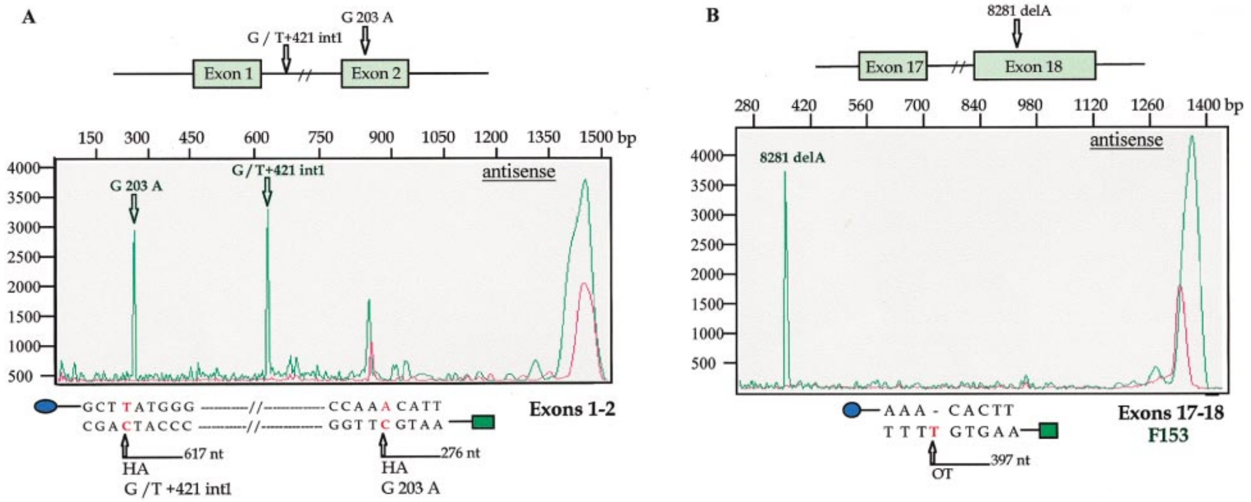

**Figure 2** Examples of fluorescent chemical cleavage profiles (both on antisense strands) obtained from large amplicons. (Panel A) Region containing exon 1, intron 1 and exon 2; total length of uncleaved DNA is 1402 nt. Peaks at 276 nt (G203A) and at 617 nt (G/T+421 int1) from the HEX fluorophore on the lower strand are observed in the C-specific (hydroxylamine, HA) reaction. (Panel B) Amplicon containing exon 17, intron 17 and exon 18. The mutation found in exon 18 is shown. An unpaired T in the noncoding strand gives rise to a cleavage peak at 397 nt from the HEX fluorophore in the T-specific reaction (osmium tetroxide, OT). Profiles shown in red in each panel represent the same strand of control samples subjected to the same CCM reaction and run on the same gel

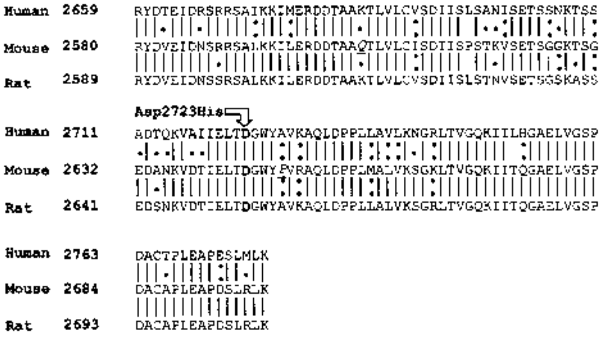

**Figure 3** Conservation across species of the Asp2723His change found in family 196. The amino acid sequence deduced from exon 18 is shown for man (Tavtigian et al, 1996), mouse (Sharan et al, 1997) and rat (McAllister et al, 1997; Genebank, accession number: NM 009765). Amino acids with similarity scores of >0.5 according to the GCG BESTFIT program are shown with double dots. Those with similarities <0.5 are shown with single dots. The Asp to His substitution, found in this patient, belongs to the latter group. Mouse residues 2605 and 2648 (both underlined) are identical to the corresponding human residues in another published mouse sequence (Connor et al, 1997)

family 196 together with two rare variants (Asn289His and Asn991 Asp). The former of these polymorphisms showed a significant deficit of heterozygosity in patients (Wagner et al, 1999). While the phase of these allelic variants could not be investigated for lack of family members, it is tempting to speculate that associations of rare alleles of *BRCA2* may sometimes result in increased predisposition to cancer. Thus, the nature of missense changes in general and in particular the effects resulting from combinations of missense changes deserve more attention also in view of the potential insights they might provide into BRCA2 function.

The contribution of *BRCA2* mutations in male breast cancer families was found to be 3/12 (25%) or 4/12 (33.3%) depending on the inclusion or not of the missense change Asp2723His. One of the truncating mutations was found in a family (number 32), which is characterized by only one case of male breast cancer and one case of female breast cancer and by a low predisposition probability (43%) of the index case. These observations suggest that the *BRCA2* gene should be screened with priority in male breast cancer families, including those with small numbers of breast cancer cases. It is also interesting to note that 2 truncating *BRCA2* mutations were found in the subgroup of 3 families with one or more cases of ovarian cancer in addition to male breast cancers (families 20 and 153 in Table 4). Our data are consistent with recent estimates of a major contribution of *BRCA2* mutations to breast cancer predisposition when at least one first-degree male relative is affected (76% in high risk families, according to Ford, 1998). On the other hand no *BRCA1* mutation was found in this group, in agreement with the low contribution of *BRCA1* to male breast cancer (Ford, 1998), although exon deletions or duplications of *BRCA1* cannot be ruled out (discussed below).

Concerning our original cohort of 59 breast ovarian cancer families, 23 truncating mutations and 4 additional likely deleterious missense mutations had been found in *BRCA1* (Stoppa-Lyonnet et al, 1997; and our unreported data). The contribution of detectable *BRCA1* mutations to this group was estimated at 46% (95% CI 33–59). It is however likely that several of the apparently *BRCA1*- and *BRCA2*-negative patients listed in Table 4 in fact had a partial *BRCA1* deletion or duplication. Screening of these patients for exon deletions or duplications is underway and a *BRCA1* rearrangement has for example been found in family 827 of Table 2 (Gad et al, in press). Recent studies have suggested that the frequency of *BRCA1* genomic rearrangements in families with breast-ovarian cancer may be equal or even greater than that of *BRCA2* mutations in coding regions (Unger et al, 2000). The 6 *BRCA2* mutations observed in this study in breast-ovarian cancer families, including 3 families in which one or more first-degree male relative(s) had breast cancer, indicate a *BRCA2* contribution of 10% (95% CI 2.5–17.5) to our original group of 59 breast-ovarian cancer families.

*© 2001 Cancer Research Campaign*

### ACKNOWLEDGEMENTS

This work was supported by grants from the Assistance Publique des Hôpitaux de Paris (No 973829, to DSL and to MT); from the Association pour la Recherche sur le Cancer (ARC, No 7282), the Fédération Nationale des Groupements des Enterprises Françaises contre le Cancer and the Fondation pour la Recherche Médicale to MT.

### REFERENCES

Connor F, Smith A, Wooster R, Stratton M, Dixon A, Campbell E, Tait TM, Freeman T and Ashworth A (1997) Cloning, chromosomal mapping and expression pattern of the mouse *Brca2* gene. *Hum Mol Genet* **6**: 291–300

Easton DF, Bishop DT, Ford D and Crockford GP (1993) Genetic linkage analysis in familial breast and ovarian cancer: results from 214 families. The Breast Cancer Linkage Consortium. *Am J Hum Genet* **52**: 678–701

Ford D, Easton DF, Stratton M, Narod S, Goldgar D, Devilee P, Bishop DT, Weber B, Lenoir G, Chang-Claude J, Sobol H, Teare MD, Struewing J, Arason A, Scherneck S, Peto J, Rebbeck TR, Tonin P, Neuhausen S, Barkardottir R, Eyfjord J, Lynch H, Ponder BA, Gayther SA, Zelada-Hedman M, et al (1998) Genetic heterogeneity and penetrance analysis of the *BRCA1* and *BRCA2* genes in breast cancer families. The Breast Cancer Linkage Consortium. *Am J Hum Genet* **62**: 676–289

Gad S, Aurias A, Puget N, Mairal A, Schurra C, Montagna M, Pages S, Caux V, Mazoyer S, Beusimon A, Stoppa-Lyonnet D. Colour bar coding the BRCA1 gene on combed DNA: a useful strategy for detecting large gene rearrangements. *Genes Chromosomes Cancer* 2001, in press

Gayther SA, Mangion J, Russell P, Seal S, Barfoot R, Ponder BA, Stratton MR and Easton D (1997) Variation of risks of breast and ovarian cancer associated with different germline mutations of the BRCA2 gene. *Nat Genet* **15**: 103–105

Griffais R, Andre PM and Thibon M (1991) K-tuple frequency in the human genome and polymerase chain reaction. *Nucleic Acids Res* **19**: 3887–3891

Marston NJ, Richards WJ, Hughes D, Bertwistle D, Marshall CJ and Ashworth A (1999) Interaction between the product of the breast cancer susceptibility gene BRCA2 and DSS1, a protein functionally conserved from yeast to mammals. *Mol Cell Biol* **19**: 4633–4642

McAllister KA, Haugen-Strano A, Hagevik S, Brownlee HA, Collins NK, Futreal PA, Bennett LM and Wiseman RW (1997) Characterization of the rat and mouse homologues of the BRCA2 breast cancer susceptibility gene. *Cancer Res* **57**: 3121–3125

Rahman N and Stratton MR (1998) The genetics of breast cancer susceptibility. *Annu Rev Genet* **32**: 95–121

Sharan SK and Bradley A (1997) Murine *Brca2*: sequence, map position, and expression pattern. *Genomics* **40**: 234–241

Stoppa-Lyonnet D, Laurent-Puig P, Essioux L, Pages S, Ithier G, Ligot L, Fourquet A, Salmon RJ, Clough KB, Pouillart P, Bonaiti-Pellie C and Thomas G (1997) BRCA1 sequence variations in 160 individuals referred to a breast/ovarian family cancer clinic. Institut Curie Breast Cancer Group. *Am J Hum Genet* **60**: 1021–1030

Tavtigian SV, Simard J, Rommens J, Couch F, Shattuck-Eidens D, Neuhausen S, Merajver S, Thorlacius S, Offit K, Stoppa-Lyonnet D, Belanger C, Bell R, Berry S, Bogden R, Chen Q, Davis T, Dumont M, Frye C, Hattier T, Jammulapati S, Janecki T, Jiang P, Kehrer R, Leblanc JF and Goldgar DE (1996) The complete *BRCA2* gene and mutations in chromosome 13q-linked kindreds. *Nat Genet* **12**: 333–337

Unger MA, Nathanson KL, Calzone K, Antin-Ozerkis D, Shih HA, Martin AM, Lenoir GM, Mazoyer S and Weber BL (2000) Screening for genomic rearrangements in families with breast and ovarian cancer identifies BRCA1 mutations previously missed by conformation-sensitive gel electrophoresis or sequencing. *Am J Hum Genet* **67**: 841–850

Verpy E, Biasotto M, Meo T and Tosi M (1994) Efficient detection of point mutations on color-coded strands of target DNA. *Proc Natl Acad Sci USA* **91**: 1873–1877

Verpy E, Biasotto M, Brai M, Misiano G, Meo T and Tosi M (1996) Exhaustive mutation scanning by fluorescence-assisted mismatch analysis discloses new genotype-phenotype correlations in angiodema. *Am J Hum Genet* **59**: 308–319

Wagner TM, Hirtenlehner K, Shen P, Moeslinger R, Muhr D, Fleischmann E, Concin H, Doeller W, Haid A, Lang AH, Mayer P, Petru E, Ropp E, Langbauer G, Kubista E, Scheiner O, Underhill P, Mountain J, Stierer M, Zielinski C and Oefner P (1999) Global sequence diversity of BRCA2: analysis of 71 breast cancer families and 95 control individuals of worldwide populations. *Hum Mol Genet* **8**: 413–423

Welcsh PL, Owens KN and King MC (2000) Insights into the functions of BRCA1 and BRCA2. *TIG* **16**: 69–74

Wooster R, Bignell G, Lancaster J, Swift S, Seal S, Mangion J, Collins N, Gregory S, Gumbs C and Mickelm G (1995) Identification of the breast cancer susceptibility gene *BRCA2 Nature* **378**: 789–792

 *© 2001 Cancer Research Campaign*
